# What Guides Organizations’ Current Dementia-Related Practices Across Four Canadian Provinces?

**DOI:** 10.3390/ijerph22050688

**Published:** 2025-04-26

**Authors:** Maria Baranowski, Nancy Jokinen, Leslie Udell, Sandy Stemp, Tracey Berman, Shahin Shooshtari

**Affiliations:** 1 Department of Community Health Sciences, Rady Faculty of Health Sciences, Max Rady College of Medicine, University of Manitoba, Winnipeg, MB R3T 2N2, Canada; 2 School of Social Work, University of Northern British Columbia, Prince George, BC V2N 4Z9, Canada; 3 NTG Canadian Consortium c/o Reena, 927 Clark Ave West, Thornhill, ON L4J 8G6, Canada; 4 L Udell Consulting, 95 Point West Dr., Winnipeg, MB R3T 5J5, Canada; 5 Reena, 927 Clark Ave West, Thornhill, ON L4J 8G6, Canada

**Keywords:** intellectual and developmental disability, dementia, dementia-related practice, disability-related practice, community living, disability-related organizations

## Abstract

We conducted a survey to learn what guides current dementia-related practice to support community-dwelling adults with intellectual and developmental disabilities who may be experiencing dementia in Canada. We invited organizations working in health, disability, or senior sectors in 4 Canadian provinces to complete an online cross-sectional survey between April and July 2023. A total of 173 people completed the survey, representing 125 unique organizations, and nearly half resided in Ontario. The most common support and services provided to adults with intellectual and developmental disabilities and their families were related to residential care, day programming, and group home living. Half of our survey respondents reported that they followed dementia-related practice guidelines. The most common guideline followed and early detection tool used were from the National Task Group on Intellectual Disabilities and Dementia Practices and the National Task Group-Early Detection and Screen for Dementia, respectively. Lack of awareness about guidelines and detection tools, challenges to implement the same, and organizational needs for future training and service provision were identified. Commitment to resources to monitor adults with IDD who may be experiencing dementia is recommended to provide meaningful support and service to them and their families.

## 1. Introduction

Adults with intellectual and developmental disabilities (IDD) are living longer [1] and, as a result, may experience age-related conditions, including dementia [2,3,4]. Based on the increasing prevalence of disability in the United States, the Neuroatypical Conditions Expert Consultative Panel examined several barriers to conducting accurate examinations of the cognitive function in aging adults with a variety of neurotypical conditions [3]. Adults born with Trisomy 21, in particular, reportedly experience increased prevalence of early onset dementia [5], and by age 65, 80% or more may be diagnosed with dementia [6]. Furthermore, adults with Trisomy 21 may experience a precipitous decline [7].

Few Canadian studies report on dementia as it affects adults with IDD [8] or their caregivers [9] or elsewhere [10]. Furthermore, Canadian provinces and territories differ in organization and delivery of their respective health and social services and data collection making it difficult to gain national level facts and statistics on this population. Sullivan et al. [11] provided primary care guidelines for use with adults with IDD in Canada, including one recommendation in reference to dementia (recommendation 32). With an apparent absence of Canadian practice guidelines to support daily, community living for adults with IDD affected by dementia and their caregivers, Canadians may rely on international research despite differences in social and healthcare systems [9].

International research highlights the need for intellectual disability support services to provide dementia education and training to family and staff caregivers, to use non-pharmacological strategies, as well as to adapt practice approaches as an individual experiences further losses as dementia progresses [12]. Another report recognized the need for research focused on community living settings for adults with intellectual disabilities affected by dementia [13].

However, we are not aware of any current Canadian specific dementia care practice guides specific to support adults with IDD living with dementia in a community. There is a vital need for such guidance as evidenced by the large numbers of support letters received from community organizations and individuals for the proposal that was submitted for funding to the Public Health Agency of Canada in 2022. The project was designed to adapt an existing set of published community support guidelines produced by the National Task Group on Intellectual Disabilities and Dementia Practices in the United States [14] from a Canadian perspective to support community-dwelling Canadians with IDD affected by dementia.

As part of the project, a survey of organizations was conducted for the purposes of collecting information from organizations across four Canadian provinces regarding their dementia care experiences and practices. It asked 35 open- and close-ended questions organized in the following sections:

- About your organization;
- About your organization’s experience;
- About your organization’s practice;
- About your organization’s learning and information needs.

The survey research was approved by a university research ethics board. The results of the survey are presented and discussed in this paper.

## 2. Materials and Methods

An online cross-sectional survey consisting of 11 close-ended and 6 open-ended questions was distributed electronically via the Survey Monkey platform to 125 organizations across four Canadian provinces, British Columbia, Manitoba, Ontario, and Saskatchewan, to collect information about their dementia care experiences and practices and future learning and information needs. Examples of close-ended survey questions included: (1) Approximately, what percentage of the adults with IDD that your organization provides support or services to are aged 40 or older? (less than 25%, between 25 and 50%, between 50 and 75%, over 75%, I don’t know), (2) Where do the adults with IDD and possible/probably dementia, who you provide support and services to, reside? (private homes, group homes, long-term care homes, other/please specify), and (3) How long has your organization provided support or services to adults with IDD who have possible/probably dementia? (less than 1 year, 1 to 5 years, more than 5 years). The main themes of open-ended questions included: (1) organizational challenges in providing dementia-related support or services to adults with IDD, and (2) learning and information needs of the organization, the organization’s leadership, and the organization’s direct support workers.

A list of organizations working in health, disability, or senior sectors within each of the four provinces was compiled and served as our initial contact list to implement the survey. Organizations were selected based on whether they provided health, dementia-, or developmental disability-related support or services to older adults with IDD, locally or provincially. Reena, founded in 1973, is a non-profit organization that provides support and services to children, adults, and seniors with developmental disabilities, physical challenges, mental health challenges, and autism spectrum disorder within a framework of Jewish culture and values. They serve close to 1000 individuals and their families through a variety of programs—residential services, day programs, family respite and social programs, counselling, and advocacy, as well as training and professional development for staff and volunteers. Reena has taken a leadership position on Seniors and Dementia, and co-chairs the Ontario Partnership on Aging and Developmental Disabilities OPADD. On 25 April 2023, Reena, in conjunction with the NTG Canadian Consortium, sent an email invitation to each organization on our original list to participate in the survey, share information about the survey, and requested that each survey respondent complete one survey per organization within a two-week period. Reena created the online survey using Survey Monkey and tracked the number of surveys distributed by tracking the number of email invitations that was sent to organizations in all four provinces. Ethics approval was sought and granted from the University of Manitoba Health Research Ethics Board.

Reena also requested that these organizations (*n* = 125) forward the survey to their networks, thereby initializing the snowball effect. A link to the online survey was included in the email invitation that was sent to organizations. The cover letter to the survey indicated that participation was voluntary and that the survey took on average approximately 10 min to complete. The survey did not ask for the name of the survey respondent, so their identity remained anonymous. Organizations were informed that they were providing consent to participate in our research study by submitting a completed survey. All participants were able to quit the survey at any time before submitting their survey, and when this occurred, their information was not saved. All responses were kept confidential. Only members of the research team had access to the raw survey data. After approximately 2 weeks, Reena sent a reminder email to all recipients of the initial email invitation who had not yet opened the email (*n* = 57) and gave them two more weeks to complete the survey. Approximately 1 week later, Reena sent another reminder to those who had not yet opened the email (*n* = 66). The survey was closed on 14 July 2023, approximately 11 weeks after it was first distributed.

Data from the completed surveys were compiled, imported, and prepared for analysis using Statistical Package for Social Sciences (SPSS), version 27. Descriptive analyses were conducted based on data collected from responses to all 11 close-ended questions on the survey. Not all of the open-ended questions were completed by all survey respondents. The most common responses provided by a variable number of respondents for each open-ended question in the survey are presented. Content analysis was conducted by one coder to explore the emerging themes based on the narrative responses to the open-ended questions included in the survey. For this reason, findings are presented as the number of responses to each open-ended question. We were unable to calculate a survey response rate as we used snowball sampling and were not able to determine the total number of recipients who received an invitation to complete the online survey on behalf of their organization. We were also unable to track the number of completed and returned surveys from the original 125 unique organizations who received the original email invitation to participate in our survey, as responses were submitted anonymously.

## 3. Results

**Figure 1:**
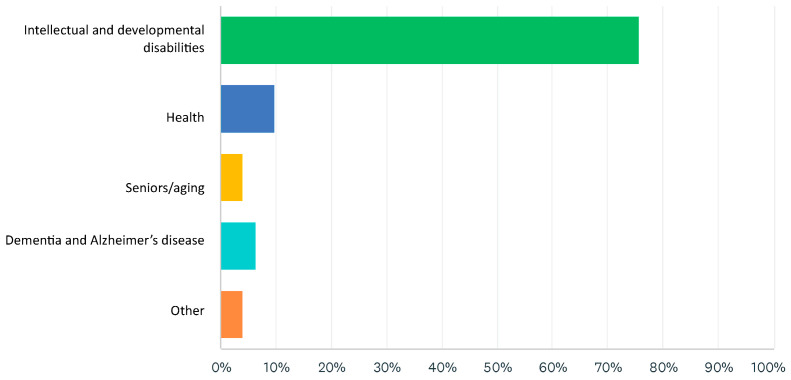
Organization by sector.

A total of 173 people completed the survey. The survey respondents represented 125 unique organizations and their networks working in health, dementia, and developmental sectors, who reported providing support and services to older adults with IDD, locally or province-wide. Over 70% (*n* = 131) of survey respondents reported that their organization worked in the intellectual and developmental disability sector (Figure 1).

Nearly half (*n* = 83) of the survey respondents resided in Ontario. There were 50 respondents from British Columbia, 25 from Manitoba, and 15 from Saskatchewan who participated in the survey. Over 40% (*n* = 72) reported that approximately 50–75% of their clientele were adults with IDD aged 40 years and older, and over 80% (*n* = 141) reported that their organization currently provided support or services to adults with IDD who also have possible/probable dementia.

Nearly 70% (*n* = 105) of survey respondents reported that they provided support or services within group homes in the community. Finally, over 80% (*n* = 128) of the survey respondents reported having provided support and services for over five years to adults with IDD who have possible/probable dementia.

### 3.1. Description of Current Organizational Dementia-Related Support and Services

Of the respondents who reported that their organization had currently provided support or services to adults with IDD who also have possible/probable dementia, most (*n* = 127) provided a description about the support and services their organization provides. The most common (20 or more responses) support and services provided related to: residential care (*n* = 48), day program (*n* = 25), and home share/group home living (*n* = 20).

Over half (*n* = 80) of the survey respondents reported that their organization followed dementia-related practice guidelines when providing support or services to adults with IDD who also have possible/probable dementia, and most (*n* = 69) described the guidelines their organizations followed (Table 1). Reportedly, the most common guideline followed was from the National Task Group on Intellectual Disabilities and Dementia Practices (*n* = 21).

However, approximately one-fifth (*n* = 33) of the survey respondents *did not know* whether their organization followed any dementia-related practice guidelines when providing support or services. When asked if their organization *was aware of* any dementia-related practice guidelines specifically for the provision of support or services to adults with IDD, nearly 40% replied either that they *did not know* (*n* = 57) and, also, that their organization *was aware* (*n* = 59). Of this latter group, over half (*n* = 28) named the National Task Group as the source of dementia-related practice guidelines. Notably, over 20% (*n* = 31) stated that they were *unaware* of any dementia-related practice guidelines that were specific to their clientele.

**Figure 2:**
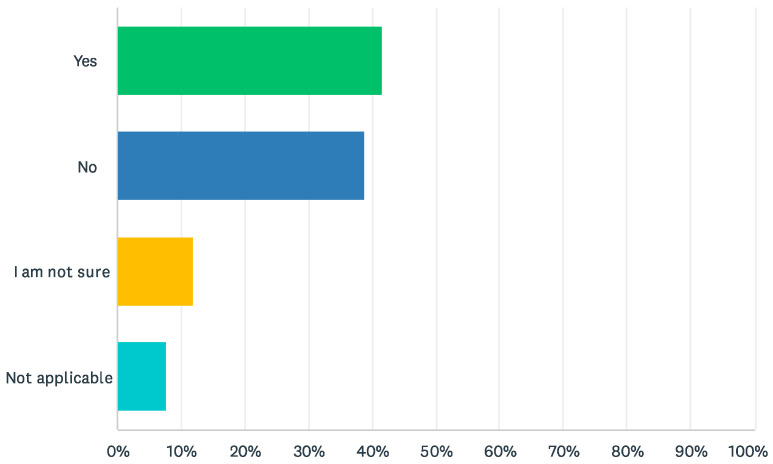
Organizational use of an early detection/screening tool.

Over 40% (*n* = 59) of survey respondents reported that their organization *did* use an early detection/screen tool on a regular basis to capture early warning signs of dementia in adults (Figure 2), and the National Task Group-Early Detection and Screen for Dementia (NTG-EDSD) [10] was the most common reported tool (*n* = 34, Table 2). Notably, almost 40% (*n* = 54) also reported that their organization *did not* use a detection/screening tool on a regular basis, and over 10% (*n* = 17) *did not know if their organization using a detection, or screening tool* (Figure 2).

The most common reported challenge reportedly experienced by organizations in the provision of dementia-related support of services to adults with IDD was a lack of education/training (*n* = 28), followed by lack of funding (*n* = 20) and lack of resources (*n* = 18). Other less frequent (*n* < 10) responses included, but were not limited to, time to detect/diagnose dementia, staff turnover and lack of access to homes and professionals.

Finally, survey respondents were asked if they had any concerns about their organization’s experience to provide support or services to adults with IDD who also have possible/probable dementia. Almost half (*n* = 74) provided responses to this question, which are listed in Table 3.

### 3.2. Description of Organizational Dementia-Related Education and Training

Over half (*n* = 79) of 145 respondents reported that their organization *does* offer dementia education or training, specifically related to adults with IDD and possible/probable dementia, to staff and/or family members. Training was most often received from the National Task Group- Canada (*n* = 26), followed by the Alzheimer Society (*n* = 12). Other less frequent (*n* < 10) responses included, but were not limited to, education/training provided by specialists, webinars, and or free public lectures.

### 3.3. Description of Potential Future Types of Organizational Dementia-Related Support and Services

Among the organizations that currently *do not* provide support or services to adults with IDD who also have possible/probable dementia (*n* = 14), most (*n* = 10) believed that their organization *will be* providing support and services in the future.

The most common reported resource organizations found to be most useful in guiding practice for the provision of support or services to adults with IDD who have possible/probable dementia was training and resources from the National Task Group-Canada (*n* = 25 out of 111 responses), followed by the Alzheimer Society (*n* = 17 out of 111 responses), and healthcare providers (*n* = 13 out of 111 responses).

Nearly half (*n* = 45 out of 104 responses) of the respondents reported that training (not specified) was the most important *need of their organization to* provide high-quality dementia-related support and services to adults with IDD. Approximately one-third (*n* = 31) of 104 respondents identified learning needs related to detection of dementia for example: (1) how to detect dementia, (2) early detection of dementia, and (3) how to differentiate between typical aging and cognitive decline. Approximately one-fifth (*n* = 21) of 104 responses identified the need for current, available, accessible, and community-based resources. Other less frequent (*n* < 15) responses included, but were not limited to, information about how to modify home environments, coping strategies, understanding of dual diagnosis of dementia and IDD, and increased funding.

Regarding *organizational leadership*, 38% (*n* = 39) of 102 responses shared that education and training about how to support and care for those experiencing dementia was needed. Other less frequent (*n* < 15) responses included, but were not limited to, a need for accurate and accessible resources, increased funding, and access to diagnostic tools and services. Finally, in relation to *direct support workers*, nearly half (*n* = 51) of 103 responses identified that education and training about what is dementia and how to support a person with dementia, and dementia and IDD, was needed. Other less frequent (*n* < 20) responses included a need for more information about best practice guidelines, how to detect dementia, early intervention, strategies to manage behavior concerns, and how to modify home environments to facilitate aging in place.

## 4. Discussion

A large number of study participants were recruited from four Canadian provinces, with nearly half of the study participants residing in Ontario (*n* = 83 out of 173). Thus, our study findings may not accurately reflect dementia-related practice in other parts of Canada. However, approximately three-quarters of our survey respondents (*n* = 131 out of 173) reported that their organization has been providing support or services to adults with IDD who also have possible/probable dementia for over 5 years (*n* = 128 out of 173 response). Surprisingly, just over half (*n* = 80 out of 151 responses) reported following dementia-related practice guidelines when providing support or services to adults with IDD who also have possible/probable dementia, and only approximately 40% (*n* = 59 out of 147 responses) were even *aware of* any dementia-related practice guidelines.

Among organizations who reported following, or who were aware of, a dementia-related practice guideline, the National Task Group (NTG) on Intellectual Disabilities and Dementia Practices was cited most often (*n* = 21 out of 69 responses and *n* = 28 out of 51 responses, respectively). Furthermore, among the 40% (*n* = 59 out of 142 responses) of organization who reported to use a detection/screening tool on a regular basis to capture early warning signs of dementia in adults, the NTG-EDSD tool was most cited (*n* = 34 out of 55 responses).

Of particular concern may be the findings that 20% (*n* = 31 out of 147 responses) were *unaware* of any dementia-related practice guidelines that were specific to their clientele; approximately one-fifth (*n* = 33 out of 151 responses) *did not know* if their organization followed any dementia-related practices guidelines when providing support or services to their clientele; and nearly 40% (*n* = 54 out of 142) reportedly *did not* use a detection/screen tool for dementia on a regular basis. More information is needed to understand why nearly half of the participating organizations in our survey did not use a detection/screen tool in order to identify barriers. It is possible that there are unknown barriers to using the detection/screening tool itself [15]. Given the reported notable lack of awareness about dementia-related practice guidelines and tools among organizations, it seems reasonable to recommend improvements to staff training and, also, better connection between organizations. Collaboration among organizations and resource sharing may facilitate consistency of high-quality service provision and also aid to increase overall awareness of developments about dementia-related guidelines and tools. For example, a train-the-trainer model of programming may be an efficient way to promote the knowledge and use of dementia-related practice guidelines and tools among organizations.

Together these findings suggest that there is a need for greater awareness of existing dementia-related practice guidelines and detection tools for adults with IDD among and within organizations. The lack of awareness among organizations about dementia practice guidelines warrants further investigation. A first step for future intervention may be to address the lack of training currently provided to staff to ensure the provision of high-quality disability- and dementia-related support and services. Commitment of resources from government to provide adequate training to paid caregivers who serve the aging population with IDD is recommended. Indeed, as is true of most disability-specific accommodations, the creation, adequate provision, and accessibility of support and services to facilitate adults with IDD also benefit those without IDD. It may also be important to further review the knowledge translation process within and among disability- and dementia- organizations to ensure the successful delivery and adoption of practice guidelines to service providers. For example, it may be necessary to share information about dementia-related practice guidelines and tools in different formats or languages [16] to satisfy the diverse learning needs of knowledge users. Furthermore, consideration of local context may be important to address barriers and increase relevance to unique organizational cultures and their direct support workers. Although their study population did not include adults with IDD, it is encouraging to learn about findings from a pilot study that reported the maintenance of wellbeing for people with dementia in homes was observed with the use of relatively inexpensive aids and adaptations [17]. Further research to explore their applicability among adults with IDD and dementia is warranted.

Although some survey respondents reported that their organizations currently offer dementia education or training that is specifically related to adults with IDD, more may be needed to address gaps identified in knowledge translation and implementation, as evidenced by our findings. We suggest continued future efforts to: (1) increase awareness of existing dementia-related guidelines for adults with IDD including the promotion of the NTG-EDSD tool, (2) expand the opportunities for NTG-Canada’s training and education workshops, (3) advocate for more resources to those providing dementia-related support and services to adults with IDD, and (4) improve collaboration among care providers. Finally, continued monitoring of adults with IDD who may be experiencing dementia is essential to ensure an accurate and meaningful understanding of what their needs are, and the needs of their care providers. Our recommendations align with those other researchers who advocated for the adaption of current residential environments into dementia-capable group home setting [18,19], by carefully considering the quality of care provision components, to determine how best to attain the right of adults with IDD and dementia to continue living in the community.

Limitations to our study include the overrepresentation of survey respondents from Ontario, Canada, which may not accurately reflect disability- and dementia-related practice in other areas. The use of snowball sampling may have also influenced sample representativeness and diversity. For example, our study cohort may not be inclusive of disability- and dementia-related service providers in smaller or remote organizations. Finally, conducting additional analyses in subsequent studies to determine correlations among key topics may provide greater insight.

## 5. Conclusions

Despite the existence of dementia-related practice guidelines for the provision of support and service to adults with IDD living in the community, more work needs to be done to ensure their acceptance and application in Canadian context. We urge policymakers to commit resources for the creation and provision of high-quality dementia-related support and services for Canadians with IDD, including training for direct support workers. We also recommend effective knowledge translation of dementia- and disability-related best practice guidelines within and among organizations that serve this population to increase awareness of and improve adoption of the same. To our knowledge, this is the first study in Canada to explore the experience of organizations providing care to adults with IDD who may experience dementia. Our findings may be used to advocate for increased attention and improved services committed to Canadians with IDD who may be experiencing dementia and to the organizations that provide these support and services, so that Canadians with IDD may safely age in place and attain their right to health and quality of life.

## Figures and Tables

**Table 1:** Dementia-related practice guidelines followed by organizations.

Dementia-Related Practice Guidelines Followed by Organizations	n
National Task Group on Intellectual Disabilities and Dementia Practices	21
Person-centered care	5
Alzheimer Society	4
Dementiability	2
Dementia screening tool, not specified	2
Advice from physician	2
Primary Care Guidelines for Physicians for Individuals with IDD	2
Community Living BC guidelines	2

**Table 2:** Dementia early detection/screening tool used by organizations.

Detection/Screening Tool	n
National Task Group Early Detection and Screen for Dementia	34
Referral for psychology/psychiatry services	5

**Table 3:** Additional concerns of organizations.

Concerns	n
Lack of appropriate and accessible resources	8
Lack of funding for services	8
Lack of training on dementia and IDD	8
Collaboration between healthcare providers in a variety of settings	6
Lack of experienced staff	6
Lack of support for aging in place	4

## Data Availability

The online survey was created and distributed by Reena and the NTG-Canadian Consortium.
